# Students’ and faculty members’ perceptions of the online component of a blended internal medicine clerkship course: a mixed-method evaluation

**DOI:** 10.1186/s12909-025-07812-5

**Published:** 2025-08-29

**Authors:** Farshad Shahkarami, Azadeh Angouraj Taghavi, Reza Hosseini Dolama, Nasim Khajavirad, Azim Mirzazadeh, Roghayeh Gandomkar

**Affiliations:** 1 Department of Internal Medicine, School of Medicine, Tehran University of Medical Sciences (TUMS), Tehran, Iran; 2 Students’ Scientific Research Center (SSRC), Tehran University of Medical Sciences (TUMS), Tehran, Iran; 3 Health Professions Education Research Center, Tehran University of Medical Sciences, Tehran, Iran; 4 Department of Medical Education, School of Medicine, Tehran University of Medical Sciences (TUMS), Roghayeh Gandomkar; No. 57, Hojatdoust St., Keshavarz Blvd, Tehran, 141669591 Iran

**Keywords:** Blended learning, Online learning, Clinical training, COVID-19 pandemic, Internal medicine, Undergraduate medical education

## Abstract

**Background:**

While online learning works well for preclinical medical education, it has limitations in clinical training. Therefore, blended learning has gained attentions as an alternative. The success of blended learning needs more attention than pure online learning or face-to-face instruction. Students and faculty members are required to manage their tasks properly using online technology disregarding the face-to-face component of blended learning. At Tehran University of Medical Sciences (TUMS), the undergraduate internal medicine course was redesigned as a blended learning course due to the pandemic. This study aimed to explore the perceptions of students and faculty members regarding the online component of the blended course, with implications for improving clinical education in low- and middle-income countries.

**Methods:**

This concurrent mixed-method evaluation focused on a 60-session online course integrated with an in-person internal medicine clerkship at TUMS during the course of four months. The study included 89 medical students and 12 faculty members as participants. Quantitative data were collected through a questionnaire assessing students’ perspectives on the online course, along with session-specific satisfaction ratings. Qualitative data were gathered through online focus group sessions with students and semi-structured interviews with faculty members, and their transcripts were analyzed in a conventional manner.

**Results:**

Students expressed high satisfaction with the course, preferring mixed online instruction and reporting positive experiences with the online platform and instructors. Faculty members found online learning to be flexible and efficient, allowing for covering a wide range of topics but faced challenges such as low student attendance, difficulties with communication, and technical issues. Overall, online learning was perceived as beneficial and capable of improvement.

**Conclusion:**

Both students and faculty members expressed satisfaction with the blended learning experience. However, some students felt that certain sessions were too long and not engaging, and the workload was a concern for some. Faculty members highlighted the need for more institutional support and recognition. Despite these drawbacks, online learning was seen as a valuable complement to traditional in-person clinical training, especially in low-resource settings. Further studies are needed to assess the long-term impact of online learning in blended clinical training, preferably with control groups.

**Supplementary Information:**

The online version contains supplementary material available at 10.1186/s12909-025-07812-5.

## Background

Medical schools experienced a forced shift toward online instruction globally with the COVID-19 pandemic [1]. Although the online approach reasonably replaced routine face-to-face methods of teaching in the pre-clinical phase, it had limitations in addressing some aspects of clinical training including providing patient contact and interaction, teaching procedural skills and clinical examination, and modeling professional socialization [2–5]. Consequently, medical students were returned to clinical placements after a period of suspension to ensure the nonstop development of clinical skills [6]. However, some schools decided to continue the experience of online learning and redesigned the clinical rotations after students return in order to utilize the positive aspects of online learning and also to diminish physical contact [7]. Since blended clinical rotations are new in undergraduate medical education programs, it is important to understand students’ and faculty members’ perceptions to inform redesign or continuation decisions.

Although there are a considerable number of studies reporting the evaluation of blended learning programs in health professions education [7], only limited numbers are related to the clinical phase of undergraduate medical education [8–12]. All of these studies considered examining blended learning as a whole, integrating face-to-face and online components. However, students and teachers, in blended learning, are required to manage their tasks properly using technology disregarding the face-to-face component. In addition, fitting the online instruction into face-to-face learning needs careful planning and more effort than designing a full online program [13]. Taking these logics into consideration, Rasheed et al. conducted a systematic review of the challenges of the online component of blended learning in all areas of education exploring students, teachers, and educational institutions’ perspectives. They concluded that these stakeholders’ experiences and behaviors vary in the online component of blended learning from entirely online courses which requires considering specific solutions for blended learning [14]. 

Despite the growing desire for designing and implementing blended learning clinical rotations, the published literature does not necessarily address a comprehensive approach to evaluation using major stakeholders’ perceptions and applying triangulation methods. Limited existing studies related to the evaluation of blended clinical rotations in undergraduate medical education were mostly applied exams to assess changes in students’ knowledge [9], attitudes [10], and performance [11, 12]. One study used a questionnaire to evaluate final-year medical students’ perceptions of learning radiology content [8]. 

Medical students went through a similar experience of suspending clinical training activities with emerging online modalities as a new approach to teaching and then returning to clinical rotations during the pandemic in Iran. At Tehran University of Medical Sciences (TUMS), we redesigned the internal medicine course, one of the most important clerkship courses, as a blended learning course with online elements in parallel with the in-person parts which the latter was held more or less as before the pandemic. Given the importance of evaluating the online component in the context of blended learning, this study aimed at exploring the perceptions of both students and faculty at TUMS regarding the online component of the blended internal medicine clerkship course employing both survey and qualitative methods. The results have implications for lower-middle-income countries to advance their clinical education by taking advantage of blended learning.

## Methods

### Design

A concurrent mixed-method evaluation was conducted to assess an online course merged with in-person internal medicine clerkship clinical rotations at TUMS in 2020.

### Setting

Internal medicine is a four-month rotation during the clerkship at TUMS. Students are divided into eight groups and go through eight two-week rotations: General internal medicine, nephrology, hematology-oncology, cardiology, endocrinology, rheumatology, gastroenterology, and pulmonology. Each medical student needs to complete all rotations, which are held at TUMS-affiliated teaching hospitals.

### Online internal medicine course

We administered a pure online internal medicine course for medical students during the pandemic while clinical training was suspended. It was our first experience with online clinical training and was a rapid and limited response to address the critical situation. We received positive feedback and reactions from the students which persuaded us to take the benefits of online learning in the consecutive cohort when clinical training was resumed. The overall goal was to cover elements of an internal medicine clerkship course that were fitted to an online setting. Consecutively, a panel of stakeholders comprising internal medicine faculty members, representatives from current and former medical students, and medical education experts was formed and reviewed the documents of the previous course including evaluation results, internal medicine rotation curriculum, and innovative reports from other medical schools around the world. Then, the panel decided on the goals, objectives, core components, format of delivery, student support, and student assessment of the new online internal medicine course.

The course included 60 online sessions that were held alongside the usual in-person internal medicine clerkship rotation. These sessions covered a wide range of important topics, using various teaching methods; such as sessions on transition to clinical setting (8 sessions), approaching common clinical complaints and clinical manifestations (13 sessions), pharmacotherapy of common diseases (8 sessions), skill development workshops for clinical reasoning, communication, medical documentation, etc. (12 sessions), introducing new topics such as geriatrics and palliative medicine (4 sessions), student morning reports (9 sessions), case presentation and question-and-answer (Q&A) sessions (6 sessions). These sessions were held in many formats including case-based learning sessions (CBL), lectures, Q&A, etc. We used both synchronous and asynchronous modalities to deliver the content. Faculty members had been previously trained in online teaching, and were oriented and supported in using the platforms during this course as well.

Mentorship was also provided by residents, M.D. graduates, interns, and peers upon students’ request. Training materials were provided to volunteer mentors, and a mentoring introductory online meeting was held. Decisions regarding further communication between mentors and mentees were delegated to the mentors and mentees, as everyone had their preferences regarding the frequency and method of communication. Students were also assessed using a variety of tools including a Multiple Choice Questions (MCQ) exam, a clinical reasoning exam, and an Objective Structured Clinical Exam (OSCE).

### In-person internal medicine course

For face-to-face component, medical students participated in visiting patients as part of a bigger medical team, in both inpatient and outpatient settings. They also participated in rounds, morning reports, journal clubs, didactic classes, and in-person workshops in the skill lab. Students were assessed with Global Rating Forms (GRFs) at the end of the rotation.

## Mixed-methods evaluation

### Participants

The study participants were 89 medical students who were going through the internal medicine clerkship course at the time and 12 faculty members who participated as tutors. The end-of-course evaluation questionnaire was administered to all students, and a diverse sample was selected from different demographic strata and invited to participate in focus groups. All faculty members were invited to participate in semi-structured interviews at the end of the course.

### Data collection

Table 1 presents data collection methods and times for quantitative and qualitative evaluation components.

**Table 1:** Data collection methods and time for quantitative and qualitative evaluation components

Evaluation component	Data collection method	Data collection time
Quantitative	Questionnaire	End of the course
	Questionnaire	End of each session
Qualitative	Semi-structured interviews with faculty members	End of the course
	Focus groups with students	End of the course
	Weekly feedback from students	Throughout the course

#### Quantitative sub-evaluation

To assess students’ perspectives on the online course, a questionnaire was developed by authors based on the existing literature and administered after the final session of the course. A panel of faculty members, students, and a medical education expert assessed the questionnaire’s face validity. The questionnaire was designed with 32 items in 8 sections: demographic and participation status, course content, teaching and learning methods, platforms, workload, assessment tools, student support services, and overall satisfaction (See Appendix file). The reliability of the questionnaire was assessed using Cronbach’s Alpha, for which the index value was 0.914. Students were also surveyed at the end of each session with two questions about their satisfaction with the session using a 5-point Likert scale (“very satisfied = 5,” “satisfied = 4,” “neutral = 3,” “unsatisfied = 2,” and “very unsatisfied = 1”). Students expressed their satisfaction with the session’s quality in general and its technical quality in particular.

#### Qualitative sub-evaluation

A total of three online focus group sessions were conducted by the first author (FS) to uncover in-depth insight into participants’ experiences with different aspects of the course. Each session consisted of five students and lasted two hours on average. In addition, we invited faculty who were involved in the course to participate in an online semi-structured interview. The faculty members were asked to describe their experience with online teaching, the advantages and challenges of online modalities, students’ learning and performance, students’ interaction, etc. The details of the interview and focus group questions are available in the Appendix.

We also gathered students’ representatives’ weekly feedback informally. All students were also provided with a Google Form to provide anonymous feedback. The research team reviewed all the feedback on a weekly basis in order to improve the quality of the course if possible.

### Data analysis

Quantitative data are reported as the mean and standard deviation. The focus group sessions and interviews were recorded, transcribed verbatim, and analyzed qualitative content analysis [15]. The analysis started with an inductive approach in which one author (FS) reviewed all transcripts and independently coded meaning units related to the research question. This step was validated with other authors (RG and AM) to ensure that the coding label demonstrates participants’ descriptions without losing the underlying meaning. Subsequently, codes were grouped deductively based on their similarities and differences into preexisting categories that were developed when the questionnaire and interviews’ questions were designed. The iterations of inductive and deductive analyses were performed and discussed with all authors until a consensus was reached on structuring the data [16].

##  Results

### Students’ perceptions

Students'perceptions were assessed by quantitative and qualitative methods, end-of-course survey, end-of-session survey, focus groups, and weekly feedback.

1. End-of-course survey: A total of 51 students (57.3%) responded to the final evaluation survey, 28 were male (55%), 22 were female (43%), and one reported non-binary (2%). A total of 44 participants were Iranian (86.3%), and the rest were international students (13.7%). Internal medicine was the first clerkship rotation for most of the participants (96.1%). As presented in Table 2, most of the respondents reported that they attended more than 75% of the sessions. The most important reasons for not attending the sessions were the inappropriate time of the sessions and students’ personal preoccupations. Most students preferred synchronous (25.5%) to asynchronous (9.8%) online instruction, and mixed online instruction (64.8%) to both modalities alone. Table 3 shows the questionnaire items with means (SD). Pharmacotherapy and approaching common clinical complaints and symptoms sessions were the most interesting ones for students (mean = 4.03 and 4.00, respectively), while students’ morning reports were the least popular (mean = 2.43). The participants were satisfied with the online platform and the communication of information (mean = 4.09 and 4.00, respectively) and using up-to-date content (mean = 4.03). The instructors’ teaching performance was reported as satisfying as well (mean = 3.66) as demonstrated in the table.

**Table 2:** Students'attendance in sessions

How many of the sessions did you attend in total?	No. of students (%) *	Which reason(s) for non-attendance apply to you?			
75% or more	37 (72.5%)	Inappropriate time of classes	28 (54.9%)	Not having access to the internet	6 (11.7%)
50% - 75%	9 (17.6%)	Personal preoccupation	24 (47.0%)	Not finding the sessions useful	6 (11.7%)
25% - 50%	2 (3.9%)	Being tired after the daily in-person education	23 (45.0%)	Not having access to an electronic device	2 (3.9%)
Less than 25%	3 (5.9%)	Preferred to watch recorded videos	10 (19.6%)	Forgetting the online sessions	2 (3.9%)

* Total No. of participants: 51

**Table 3:** Students’ satisfaction with the components of the online internal medicine course

Items	Mean (SD)
The content was congruent with my previous level of knowledge.	3.7 (0.67)
The content was congruent with the educational methods used to deliver it.	3.74 (0.79)
The content and educational materials were up-to-date.	4.03 (0.59)
The content was engaging and appealing to me.	3.33 (0.79)
The topics of different sessions were coherent and relevant together.	3.52 (0.85)
It was possible to effectively interact with my peers and the instructors.	3.98 (0.58)
The course’s overall planning was appropriate.	3.33 (0.79)
The instructors were competent at online teaching.	3.66 (0.71)
“Students’ morning report” sessions were useful and satisfactory.	2.43 (0.98)
“Transition to clinical practice” sessions	3.58 (0.96)
“Approaching common diseases and clinical manifestations” sessions were useful and satisfactory.	4.00 (0.87)
“CBL” sessions were useful and satisfactory.	3.82 (0.99)
“Pharmacotherapy of common internal medicine diseases” sessions were useful and satisfactory.	4.05 (0.83)
“Clinical reasoning” workshops were useful and satisfactory.	4.03 (0.93)
“Q & A sessions” were useful and satisfactory.	3.11 (1.01)
The quality of the main platform (3B) was satisfactory.	4.09 (0.72)
The course workload was reasonable.	3.23 (1.01)
The duration of sessions was appropriate.	3.54 (0.83)
Technical support services were available if needed.	3.60 (0.89)
Scientific support and advice were available if necessary. (by the instructors and mentors)	3.58 (1.04)
The communication of course information was satisfactory.	4.00 (0.69)
Recorded videos of the sessions were readily available.	3.76 (0.90)
It was possible to effectively give feedback to the course organizers.	3.47 (0.96)
Mentors were available throughout the course for guidance.	3.53 (0.98)
The online course helped me improve my clinical reasoning skills.	3.64 (0.79)
The online course helped me improve my communication skills.	2.94 (0.98)
The online course helped me improve my medical knowledge.	3.62 (0.77)
The quizzes helped reduce my pre-exam stress.	3.50 (1.00)
The quizzes helped improve my learning and motivation.	3.37 (1.01)
The online course was overall satisfactory.	3.62 (0.82)
2. End-of-session survey: Table 4 shows the results of students' general satisfaction and their perceptions on the technical quality of each session with the number of respondents for each session (range = 31-57). Most sessions scored more than 4 out of 5 on average. Clinical reasoning and pharmacotherapy sessions were the most satisfying sessions (mean= 4.76 and 4.74, respectively). Technical quality of all sessions was scored more than 4 out of 5 on average, except for two sessions.

**Table 4:** Result of satisfaction surveys at the end of each session

**Session topic**	**General satisfaction**		**Audio-visual quality**	
	**N**	**Mean (SD)**	**N**	**Mean (SD)**
Introduction to palliative medicine (pain management)	45	2.84 (1.27)	N/A	N/A
Introduction to palliative medicine (management of GI symptoms)	39	2.02 (1.36)	35	2.05 (1.25)
Introduction to geriatrics and elderly health	47	2.91 (1.29)	N/A	N/A
Introduction to common geriatric syndromes	47	2.29 (1.33)	N/A	N/A
Clinical reasoning session (CBL) – 1	55	4.40 (0.93)	53	4.69 (0.6)
Clinical reasoning session (CBL) – 2	39	4.76 (0.53)	34	4.79 (0.47)
Clinical reasoning session (CBL) – 3	45	4.51 (0.72)	43	4.55 (0.62)
Clinical reasoning session (CBL) – 4	45	4.66 (0.6)	44	4.4 (0.87)
Approaching common clinical complaints and symptoms – 1	58	4.50 (0.82)	58	4.67 (0.88)
Approaching common clinical complaints and symptoms – 2	52	4.32 (0.8)	44	4.68 (0.6)
Approaching common clinical complaints and symptoms – 3	40	4.47 (0.67)	38	4.65 (0.48)
Communication skills workshop (Dealing with angry patients)	45	4.31 (0.77)	42	4.47 (0.59)
Communication skills workshop (Delivering bad news)	31	4.64 (0.71)	32	4.78 (0.49)
Approaching anemia (CBL)	45	4.48 (0.72)	42	3.71 (1.08)
Approaching dyspnea (CBL)	42	4.26 (1.06)	N/A	N/A
Interpretation of CXR	54	4.5 (0.77)	55	4.76 (0.46)
Student case presentations	36	4.63 (0.59)	34	4.82 (0.38)
Personal protection principles during the COVID-19 pandemic	39	4.30 (0.89)	57	4.54 (0.65)
Principles of medical documentation (CBL) −1	57	4.54 (0.65)	58	4.46 (0.68)
Principles of medical documentation - History taking (CBL) – 2	47	4.27 (0.79)	47	4.36 (0.96)
Principles of medical documentation - Progress note (CBL) – 3	54	4.38 (0.87)	52	4.01 (1.17)
Pharmacotherapy of common diseases (Asthma)	44	4.63 (0.61)	44	4.63 (0.53)
Pharmacotherapy of common diseases (Diabetes)	34	4.61 (0.74)	35	4.82 (0.38)
Pharmacotherapy of common diseases (Dyslipidemia)	41	4.61 (0.77)	42	4.57 (0.66)
Pharmacotherapy of common diseases (IBS)	35	4.74 (0.50)	34	4.79 (0.41)
Pharmacotherapy of common diseases (Pain management)	41	4.63 (0.69)	41	4.60 (0.66)
Transition to clinical setting and clinical learning skills	39	3.94 (0.72)	37	4.45 (0.73)

*N/A *Not Available, *CXR* Chest X-ray, *GI* Gastrointestinal, *IBS* Irritable Bowel Syndrome
3. Focus group sessions: Fifteen students, eight females, and seven males, participated in these sessions. A total of 49 subcategories were identified and categorized into six categories (Table 5):

**Table 5:** Codes and Categories extracted from focus groups with students

Quotes	Sub-categories (No. of Initial Codes)	Categories
*“… documentation and history-taking workshops were efficient and pharmacotherapy sessions were really helpful in in-person rotations…”*	General satisfaction (18)Comparison with Previous e-learning Experiences (5)Course efficiency (14)The usefulness of theoretical content covered in the course (3)The usefulness of the skills covered in the course (20)	Satisfaction and Efficiency
*“… E-learning was really good and necessary and it should be used even after the COVID-19 pandemic. This course was much better than the other ones we’ve had, and it showed me the opportunities that e-learning can provide and its potential…”“Some sessions must be optional; to provide the students with the right to choose the classes that they think they need to participate in…”*	General attitudes (19)Change of attitudes (5)Comparison to in-person education (3)Time management benefits (3)Possibility of giving effective feedback (2)Flexibility of the program (3)Teacher-Student interactions (1)Instructors’ performance (4)Other strengths (4)Other weaknesses (4)Future of online learning (9)Suggestions for improving the quality of e-learning (25)	Attitudes Towards Online Learning
*“Interactive sessions were really good and useful... Their content sticks to my mind and I can remember what I had learned several months after the sessions… I’ve finally learned what I need as a general practitioner.”“Teaching geriatrics and palliative medicine was a good idea… but it would’ve been better if the sessions were shorter and more practical…”*	Curriculum (26)Logical order of the course topics (7)Overlapping topics and repetitiveness (9)Geriatrics sessions (2)Palliative medicine sessions (2)Geriatrics & Palliative medicine (Unspecified) (18)CBL sessions (14)Peer mentoring: strengths and weaknesses (11)Peer mentoring: reasons for not using it (10)References (5)	Course Curriculum
*“… Pharmacotherapy sessions were the best part of the program. The “approaching common clinical manifestations” classes were useful too since they were based on practical approaches and guides... The interactive nature of these sessions was their strength.”“Students’ morning reports were good to learn more about history taking and presentation skills; but the cases were not educational enough…”*	“Pharmacotherapy” sessions (19)“Approaching common manifestations and diseases” sessions (12)CBL sessions (12)Student morning reports (19)Clinical reasoning workshops (6)“Abnormal physical examination findings” sessions (5)Communication skills workshops (8)“Transition to clinical setting” sessions (11)	Teaching methods and topics of the sessions
*“… 3B was the best online platform I’ve used so far.”*	Online Platforms (14)Strengths & features (3)Weaknesses (4)Privacy issues (1)Comparison of platforms (3)E-poll (6)	Platforms
*“…[Professors] didn’t even know our names to evaluate us individually! They often gave the whole group a similar score.”*	Students’ assessment (7)Faculty’s evaluation of students (12)End-of-rotation assessments (GRFs) (4)Feedback (4)MCQ exam (10)Open-book exam (8)OSCE (12)Clinical reasoning exam (9)	Students’ Assessment

  - Satisfaction and efficiency: Most participants were satisfied with the course. The advantages of this online course made it significantly better than their previous experiences. Interactive teaching methods, suitable platforms, and relevant content were among the advantages. This online course was considered a proper complement to the program and helped increase students'competencies. Students also believed that the in-person component is still the main part of the clerkship, and the online component should complement it, as the online course helped them in their rotations. They also believed that physical examinations and procedural skills must be taught in-person, and were somewhat neglected in their clinical education.
  - Attitudes towards online learning: Most students stated that the course changed their attitudes toward online learning positively, and they preferred it to in-person instruction due to the flexibility of the program, the possibility of giving and receiving effective feedback, and easier access to professors. On the other hand, having too many sessions and poor quality of the content of some of the lectures were mentioned as the weaknesses of the online course. Albeit, some participants attributed weaknesses to clinical training and lectures in general, not to their online nature. Most students regarded online education positively, and they preferred it to be continued after the pandemic.
  - Course Curriculum: The course plan was generally regarded as useful, practical, and satisfying by participants. However, some criticism and suggestions were provided regarding the incoherence of topics, the excessive length and number of sessions, and the limited use of asynchronous online instruction. To improve the course quality, several suggestions were made, including providing an offline case bank, reducing the number of sessions, and revising the topics. Students also mentioned that palliative medicine and geriatrics sessions were too long and lecture-based and suggested more practical points. However, CBL sessions were interactive and useful, allowing them to be well-received by students. The peer mentoring program proved to be controversial, with some participants finding it beneficial and others finding it unnecessary. For example, some mentors shared clinical experiences with their mentees, helping them learn about physical examinations and procedures better. However, some students had prior negative experiences with peer mentoring and were reluctant to reach out to their mentors.
  - Teaching methods and topics of the sessions: Most participants were satisfied with all sessions except student morning reports and communication skills workshops. The participants considered the pharmacotherapy sessions to be the most satisfactory and the best part of the online course. They also believed that teaching the clinical manifestations of prevalent diseases was necessary. CBL sessions were described as attractive due to the experience of teamwork, while communication skills workshops were theoretical and lecture-based and were not as appealing. The students’ morning reports were also not satisfactory due to inappropriate time and content.
  -  Platforms: This program used a variety of online platforms, including BigBlueButton (3B) which is a synchronized online meeting software, Google Forms, and e-Poll (a free poll and questionnaire platform). The strengths of 3B were mentioned as an appropriate teamwork space and polling capabilities. Meanwhile, other platforms covered its weaknesses (e.g. Google Forms were used for short-answer quizzes.)
  - Students’ Assessment: Students considered using different methods for learning assessment to be positive and better than previous experience in other rotations. Students believed that OSCEs and clinical reasoning evaluations were acceptable. However, they preferred that the OSCE not be online, so that clinical procedures could be assessed appropriately. Many students found the MCQ exam challenging because of the high content volume. Students also provided several comments on the in-person component of assessment and mentioned that professors'evaluations were generally subjective and not related to GRF items.
4. Student feedback: The participants'feedback focused on three main issues: satisfaction with CBL sessions, language barriers leading to communication difficulties for international students, and class time scheduling. These feedbacks were presented to the course organizers to be considered in course planning.

### Faculty members'perceptions

Of 12 faculty members, five (41.6%) agreed to participate in the semi-structured interviews. Four were internists and one was a clinical pharmacist. A total of 67 sub-categories organized into seven categories were extracted (Table 6):

**Table 6:** Codes and sub-Categories extracted from interviews with faculty members

Quotes	Sub-categories (no of initial codes)	Categories
*“…Those students who really want to learn will interact with you, whether the class is in-person or online…”* *“These [Students] were knowledgeable and different from the ones who didn’t have this course last semester…”*	Satisfaction (9) Performance (5) Education quality (4) Comparison to in-person education (10) Learning experiences (4) Educational goals (2) Students’ assessment (6) Students’ competencies (6) IKHC vs. other teaching hospitals (5)	General satisfaction and performance
*“… Our solution was to ask questions and use the chat box or polls to communicate and interact with the students.”*	Active participation (15) Communication issues (9) Communication tools (6) Interaction with students (27) Body language and eye contact (7)	Communication with the students
*“There was no official technical support either from the hospital or the university.”* *“Unlike many platforms, 3B is open-source and free for unlimited use…”*	Internet connection (7) Online platforms (2) Problems (2) Tools (8) Technical support (10) Technical skills (3) 3B (9) Trittapp (2) Sky room (4) Zoom (2) Google Meet (2)	Platforms and Technical issues
*“Now it’s not necessary to spend a couple of hours every day for commuting…”*	Strengths (30) Time consumption (17) Financial costs (2) Commuting (1) Physical environment (6) Flexibility (4) Accessibility (5)	Advantages and Strengths of online learning
*“The most important challenge is [lack of] socializing and [learning] social skills. Because practicing medicine needs face-to-face communication with patients…”*	Challenges (13) Non-participating students (2) Government censorship and filtering (2) Socialization limitations (1) Declining discipline (1) Weaknesses (8)	Challenges of online learning
*“…Students’ satisfaction and gratitude was the best reward for me...”* *“…Interactive sessions were well-received by the students according to their positive feedback.”*	Educational content and materials (11) Teaching methods (6) Difficulties (4) Burden and work overload (3) Coordination of sessions (5) Possibility of giving feedback (2) Flexibility (2) Implementation issues (7) Sessions’ topics (4) Blended learning (4) Synchronous learning (4) Asynchronous learning (7) Acknowledgment of efforts (9) Providing feedback (6) Compensation and reward system (4) Expectations (2) Scientific presentations (1)	Course implementation
*“Blended programs are good and we can use e-learning even after the COVID-19 pandemic…”* *“Practically any theoretical subject can be taught online.”* *“I didn’t use to like online learning, but when I saw in this course that it can have good quality, I changed my mind about it.”* *“…I could listen to students’ discussions in breakout rooms [in 3B] and it was interesting for me to know how they thought…”* *“[e-learning] has significantly improved the quality of education in the internal medicine [clerkship] program. It enabled us to teach what we could not before…”*	General attitudes (27) Acceptance (7) Resistance (10) Normalization (2) Feelings on e-learning (9) Trust issues (3) Faculty development (10) Adapting to new environments (6) Previous experiences with e-learning (8) Similar experiences (4) Resilience (3) Future of online education (13)	Attitudes towards online learning

- General satisfaction and performance: Faculty members were satisfied with the course quality and their own performance. By providing a more flexible schedule, hybrid education enabled them to allocate more time to their educational responsibilities and cover different topics and all key objectives, therefore making hybrid education more desirable than in-person education alone. Low attendance of students, inactive participants, difficulties in interacting with international students, and lack of face-to-face communication were also mentioned as challenges. The instructors believed that the students performed well in their clinical rotations and used acquired skills.
- Communication with the students: Communication with learners in online sessions was more challenging than communication in in-person instruction due to the inability to establish effective communication through body language, eye contact, and tone and due to students'low engagement and non-attendance. Proper interaction with students and familiarity with online platforms could affect instructors'perceptions and satisfaction.
- Platforms and technical issues: Connecting to the internet was the most problematic issue for students and faculty. On some occasions, there was a lack of appropriate technical support, although these problems were less serious than they were previously experienced. Being open-source and providing breakout rooms, polls, and tools for monitoring students’ performance were mentioned as platform strengths.
-  Advantages and strengths of online learning: The use of online education could reduce commuting and physical environment utilization and increase flexibility, thereby saving time and money. In addition, future courses can be held using the educational materials provided in this course.
- Challenges of online learning: Educators also found e-learning challenging due to poor interaction, inactive participants, the need for new skills, and communication issues. A potential lack of social skills in the future was also a concern.
- Course implementation: The program was mostly implemented as designed; however, some modifications were made based on feedback from the students and instructors. Flexibility was mentioned as an advantage of e-learning in general and this course. Most instructors preferred blended learning over online or in-person learning and synchronous sessions over asynchronous sessions. They also believed interactive classes such as CBL sessions to be the most beneficial for students. There was no institutional reward system, and the instructors did not receive any official compensation, acknowledgment, or feedback of any kind. Faculty members were mostly self-motivated to improve their performance.
- Attitudes toward online learning: While this course displayed the strengths of e-learning and positively changed the instructors'opinions, they still held a wide range of opinions. Most instructors thought that blended learning should continue after the pandemic. E-learning advocates felt it was a proper way to teach clinical topics, while opponents found it unsatisfactory due to a lack of interaction with students. This program changed instructors’ opinions about e-learning. Most thought it should continue after the pandemic, and this course could be a successful model for future programs.

## Discussion

The majority of previous studies evaluated pure online instruction administered during the whole educational program such as the undergraduate medical education program or during the preclinical phase. The results of these studies identified that online learning offers substantial possible benefits over more traditional instruction methods as it has the potential to disseminate large amounts of information and provide valuable educational resources to great numbers of students [17, 18]. This study applied online learning to a blended internal medicine clinical training and evaluated medical students’ and faculty members’ perspectives. The findings demonstrated that the use of online learning alongside in-person instruction improves clinical training.

Our findings demonstrated that students were satisfied with the online course since it covered several knowledge-based sessions, particularly pharmacotherapy. Students also agreed that new and up-to-date subjects were provided through the online course. Students showed positive reactions to clinical reasoning and approaching common clinical complaints and symptoms sessions. This satisfaction may be due to the reason that we developed our course based on the needs of the students and selected the topics that were overlooked in the usual clinical training. Faculty members believed that online learning enabled them to cover different topics and all the key objectives of the course. The findings reveal that online learning can be complementary to traditional clinical rotations particularly in internal medicine in terms of supplying it with new necessary subjects and clinical skills. However, students were not satisfied with all the sessions. For instance, they mentioned in the focus group sessions that palliative medicine and geriatrics sessions were too long and not engaging. In terms of session format, students valued CBL sessions unlike morning reports and some communication skills workshops. This finding reveals that interactive teaching techniques and formats are important for the success of online learning. Also, the students received the sessions on pharmacotherapy of common diseases very well, since it was engaging and they found its content helpful in their practice. Our findings are in line with implementing blended learning for medical students in a community preceptorship. The researchers found that the online interactive modules were well accepted by students, regardless of the additional time students spent completing them [19]. 

One concern of students in the current study was the overload of instruction due to the high number and duration of online sessions. Similarly, findings of a study in Indonesia indicated that medical students considered the workload of blended learning quite heavy so it needed good time management [20]. Our participants believed that since online modalities provide the opportunity to have instruction out of the routine clinical teaching time, it influences their personal schedule and life. As noted by Rasheed et al., the challenges of regulating learning in blended learning are more serious than in sole online learning. This has implications for course directors, first, to pay attention to the management of the syllabus of the blended learning course and, second, to consider strategies for enhancing the self-regulation of students alongside the course. They reported in their systematic review many challenges for the online component of blended learning in all areas of education. Most challenges were related to technological issues including teachers’ and students’ technological literacy, technological operation and provision, and students’ isolation [14]. These findings are not completely aligned with the results of our study. Although faculty members expressed some concerns about the students’ involvement in the sessions, we found that students and faculty members were satisfied with the technological aspects of the online course. Students scored the transferring of information on the platform very well. The difference between our findings and Rasheed et al. may be because they reported studies from all spectrums of education and university students may have fewer problems with technology [14]. Given the contradictory findings, it is recommended to conduct review studies on online learning in clinical training to identify the areas of strengths and weaknesses [21–23]. 

We provided online peer mentoring throughout this course. Students’ opinions were heterogeneous; some expressed great satisfaction and maintained close relationships with mentors. Meanwhile, other students reported that they were not able to get the most out of their mentors. These findings are consistent with previous research studies that though peer mentoring is perceived to be a vital component of academic medicine and personal development, its impact varies widely, and the body of evidence to support such notions is not strong [24–27]. 

Unfortunately, the faculty members stated that they did not receive any encouragement from medical school and these activities are not reflected in their evaluation and promotion. The participants stated that their main source of motivation was the satisfaction they received from individual learning and the satisfaction of students and their feedback. Based on the study by Elshami et al., institutional support and organizational policy can contribute to faculty satisfaction. The faculty reported dissatisfaction with higher workload, longer preparation time, technical problems, lower student participation in online discussions, and their ability to provide feedback during e-learning [23]. 

Despite some drawbacks, we found that students and faculty were generally satisfied with the online course. Students preferred online learning over traditional teaching and consistent with the literature, most of the faculty believed that online learning should be an integrated part of the future of medical education and should not be abandoned with the end of the COVID-19 pandemic [20, 21]. Our participants emphasized online learning as a complementary to in-person clinical training in knowledge-based subjects. Limited existing research has indicated that e-learning was less effective than traditional teaching for medical students in the clinical phase in areas such as balancing theoretical and practical experience and professional development [28]. It is interesting to explore the effect of online learning component of blended learning in clinical context (where role model observations and interactions are very important in learning) on the development of competencies for professional attitudes and behaviors in future studies.

According to previous studies, students’ and faculty members’ perspectives on online learning are significantly influenced by their perceptions of the challenges associated with this mode of instruction. If stakeholders perceive online learning as useful and easy to use, they are more likely to continue using it, whereas if they perceive it as challenging and difficult to use, they are less likely to continue [29–31]. Our findings are promising for low-resource countries that can benefit from blended learning potentials in clinical training with no serious concerns about technology issues.

### Limitations

There are several limitations to this study. First, this study denotes the evaluation of one clinical course. Although internal medicine is a fundamental course in undergraduate medical education, the generalizability of our findings is limited. Second, we did not report the outcome of the course. It is recommended to conduct further evaluation of blended clinical teaching with the triangulation of process and outcome assessment. Another limitation is that we explored the students and faculty members’ perceptions about the online component of the internal medicine rotation. Further research is recommended to evaluate the in-person component (the clinical experience of students) alongside the online learning. As an another limitation, since this study was conducted during the pandemic when social distancing was mandatory, we were not able to hold interviews or focus group sessions in person which could have influenced the data. Lastly, some students did not respond to the final survey and refused to participate in the focus group sessions. This might have resulted in an underrepresentation of their points of view. The same was true regarding the instructors who did not participate in the interviews.

##  Conclusions

This study evaluated the perceptions of medical students and faculty regarding an online component of a hybrid internal medicine clerkship course. Generally, both faculty members and medical students were satisfied with the online learning experience. We found that in case of appropriate planning and using interactive teaching methods, online learning modalities can be a suitable complementary to face-to-face learning in clinical rotations. However, the overload of sessions was a drawback in our online course. Some faculty members needed help adapting to the new learning modalities and could have used more institutional support. Our findings revealed that internal medicine clerkship is well-suited to the electronic mode of delivery in low-middle countries. Future studies can investigate the short and long-term effects of online learning.

## Supplementary Information

Supplementary Material 1.

## Data Availability

The datasets used and analyzed in this study are available from the corresponding author upon reasonable request.

## Abbreviations

3B: BigBlueButton
CBL: Case-based Learning
COVID-19: Coronavirus Disease 2019
CXR: Chest X-Ray
E-learning: Electronic Learning
GI: Gastrointestinal
GRFs: Global Rating Forms
IBS: Irritable Bowel Syndrome
IKHC: Imam Khomeini Hospital Complex
MCQ: Multiple Choice Question
N/A: Not Available
OSCE: Objective Structured Clinical Examination
Q & A: Question and Answer
SD: Standard Deviation
SSRC: Students’ Scientific Research Center
TUMS: Tehran University of Medical Sciences
