# Supplementary material for: Students’ and faculty members’ perceptions of the online component of a blended internal medicine clerkship course: a mixed-method evaluation
Source: BMC Med Educ. 2025 Aug 29;25:1221. doi: 10.1186/s12909-025-07812-5 (PMC12395719; doi:10.1186/s12909-025-07812-5)
Supplement: Supplementary file 1 — Supplementary Material 1. [file 12909_2025_7812_MOESM1_ESM.docx]

**The end-of-course evaluation questionnaire** (**Students’ satisfaction with the components of the online internal medicine course)**

Dear students, Hello.

Now that your internal medicine clerkshipcourse has ended, it is a great opportunity for usto know your opinion about the effectiveness and success of this course. We hope that by taking your perspectives into account we can identify the strengths and weaknesses of this course and organize future courses with better quality. Please notethat the purpose of this questionnaire is tospecifically evaluate **the online component** of the internal medicine course, and the evaluation of the on-person component of the coursethat you went through in the hospitals will be done elsewhere.

This questionnaire has been designed by the internal medicine departmentwith the aim of measuring your satisfaction and collecting your opinions. We will be grateful if you help us improve the quality of education by completing this questionnaire. This questionnaire includes 33 questions and itwill take approximately 10 minutes to complete. It is anonymous and there is no need to submit your name, student number, or any other identifying information.

This questionnaire is part of a more comprehensive project on evaluating the online internal medicine clerkship course. This research project is approved by the research ethics committee of TUMS, and registered with the IRB code of IR.TUMS.IKHC.REC.1400.032. Participation is completely voluntary. The results of this evaluation might be published in a peer-reviewed journal.

We appreciate your kind support.

Sincerely, the course evaluation team, November 2021

**Part 1: Demographic Information**

- Gender:
  - Male
  - Female
  - Other (non-binary)
  - Do not want to declare
- Nationality:
  - Iranian
  - Citizens of other countries (International student)
- Was this course your first clerkship experience?
  - Yes
  - No
- Of the 60 sessions, how many did you attended approximately?
  - 46 sessions or more (more than 75%)
  - 31 to 45 sessions (between 50% to 75%)
  - 16 to 30 sessions (between 25% to 50%)
  - 15 sessions or less (less than 25%)
- Which of the following describes the reason(s) for your non-attendance in the online sessions? (Choose all that apply)
  - Not having access to the internet
  - Not having access to electronic device (such as a laptop, tablet, or smart phone)
  - Personal preoccupations
  - Preferred to watch the recorded videos of the sessions
  - Not finding the sessions useful
  - Inappropriate time of classes
  - Being tired after the daily in-person education
  - Other issues (Explain shortly)

**Part 2: Effectiveness of the course content and organization**

In the next 5 questions, please choose how much you agree or disagree with each statement.

- The content was congruent with my previous level of knowledge.
  - Strongly disagree
  - Disagree
  - Neutral
  - Agree
  - Strongly Agree
- The content was congruent with the educational methods used to deliver it.
  - Strongly disagree
  - Disagree
  - Neutral
  - Agree
  - Strongly Agree
- The content and educational materials were up-to-date.
  - Strongly disagree
  - Disagree
  - Neutral
  - Agree
  - Strongly Agree
- The content was engaging and appealing to me.
  - Strongly disagree
  - Disagree
  - Neutral
  - Agree
  - Strongly Agree
- The topics of different sessions were coherent and relevant together.
  - Strongly disagree
  - Disagree
  - Neutral
  - Agree
  - Strongly Agree

**Part 3: The effectiveness of teaching-learning activities**

In the next 10 questions, please choose to what degree you agree or disagree with each statement. If you have not participated in the sessions in question, please leave the question unanswered.

- It was possible to effectively interact with my peers and the instructors.
  - Strongly disagree
  - Disagree
  - Neutral
  - Agree
  - Strongly Agree
- The course’s overall planning was appropriate.
  - Strongly disagree
  - Disagree
  - Neutral
  - Agree
  - Strongly Agree
- The instructors were competent at online teaching.
  - Strongly disagree
  - Disagree
  - Neutral
  - Agree
  - Strongly Agree
- “Students’ morning report” sessions were useful and satisfactory.
  - Strongly disagree
  - Disagree
  - Neutral
  - Agree
  - Strongly Agree
- “Transition to clinical practice” sessions
  - Strongly disagree
  - Disagree
  - Neutral
  - Agree
  - Strongly Agree
- “Approaching common diseases and clinical manifestations” sessions were useful and satisfactory.
  - Strongly disagree
  - Disagree
  - Neutral
  - Agree
  - Strongly Agree
- “CBL” sessions were useful and satisfactory.
  - Strongly disagree
  - Disagree
  - Neutral
  - Agree
  - Strongly Agree
- “Pharmacotherapy of common internal medicine diseases” sessions were useful and satisfactory.
  - Strongly disagree
  - Disagree
  - Neutral
  - Agree
  - Strongly Agree
- “Clinical reasoning” workshops were useful and satisfactory.
  - Strongly disagree
  - Disagree
  - Neutral
  - Agree
  - Strongly Agree
- “Q & A sessions” were useful and satisfactory.
  - Strongly disagree
  - Disagree
  - Neutral
  - Agree
  - Strongly Agree

**Part 4: The Online Platform**

In the next question, please choose to what degree you agree or disagree with the statement about the online platform used in this course.

- The quality of the main platform (3B) was satisfactory.
  - Strongly disagree
  - Disagree
  - Neutral
  - Agree
  - Strongly Agree

**Part 5: The course workload and assessment tools**

In the next four questions, please choose to what degree you agree or disagree with each statement.

- The course workload was reasonable.
  - Strongly disagree
  - Disagree
  - Neutral
  - Agree
  - Strongly Agree
- The duration of sessions was appropriate.
  - Strongly disagree
  - Disagree
  - Neutral
  - Agree
  - Strongly Agree
- The quizzes (online mock exams) helped reduce my pre-exam stress.
  - Strongly disagree
  - Disagree
  - Neutral
  - Agree
  - Strongly Agree
- The quizzes (online mock exams) helped improve my learning and motivation.
  - Strongly disagree
  - Disagree
  - Neutral
  - Agree
  - Strongly Agree

**Part 6: Student Support Services**

In the next six questions, please choose to what degree you agree or disagree with each statement.

- Technical support services were available if needed.
  - Strongly disagree
  - Disagree
  - Neutral
  - Agree
  - Strongly Agree
- Scientific support and advice were available if necessary. (by the instructors and mentors)
  - Strongly disagree
  - Disagree
  - Neutral
  - Agree
  - Strongly Agree
- The communication of course information was satisfactory.
  - Strongly disagree
  - Disagree
  - Neutral
  - Agree
  - Strongly Agree
- Recorded videos of the sessions were readily available.
  - Strongly disagree
  - Disagree
  - Neutral
  - Agree
  - Strongly Agree
- It was possible to effectively give feedback to the course organizers.
  - Strongly disagree
  - Disagree
  - Neutral
  - Agree
  - Strongly Agree
- Mentors were available throughout the course for guidance.
  - Strongly disagree
  - Disagree
  - Neutral
  - Agree
  - Strongly Agree

**Part 7: Overall Satisfaction**

In the next four questions, please choose to what degree you agree or disagree with each statement.

- The online course helped me improve my clinical reasoning skills.
  - Strongly disagree
  - Disagree
  - Neutral
  - Agree
  - Strongly Agree
- The online course helped me improve my communication skills.
  - Strongly disagree
  - Disagree
  - Neutral
  - Agree
  - Strongly Agree
- The online course helped me improve my medical knowledge.
  - Strongly disagree
  - Disagree
  - Neutral
  - Agree
  - Strongly Agree
- The online course was overall satisfactory.
  - Strongly disagree
  - Disagree
  - Neutral
  - Agree
  - Strongly Agree

**Part 8: Open-ended questions**

The following three open-ended questions are designed to gather your feedback and opinions that were not covered in the previous questions. Feel free to share your opinion about the online course, whether positive or negative. We welcome all your feedback, suggestions, and ideas to improve the quality of this educational program.

- In your opinion, what were the strengths of this online course?
- In your opinion, what were the weaknesses of this online course?
- What suggestions do you have to improve the quality of this course or similar courses in the future?

**We are sincerely grateful for your time and cooperations.**

If you have any concerns or questions, you can contact the course’s organizer (Dr. Nasim Khajavi Rad) or the lead researcher. (Dr. Farshad Shahkarami)

**Focus group questions**

1. In general, how do you feel and what do you think about the online component of the internal medicine clerkship program? Please explain.
2. What was the best and worst thing about this course for you? What do you think are the strengths and weaknesses of this course? Please explain.
3. In this course, you experienced sessions with different topics and educational methods. What do you think about each type of these sessions? Please explain.
4. How were the sessions related to each other? (for example, in terms of overlap and coherence). Please explain.
5. In this course, some new ideas were tested to improve the quality of your education (such as mentoring, Q&A sessions, geriatric and palliative medicine sessions) What do you think about each of these ideas? What are the ways to improve their quality? Please explain.
6. What advantages or disadvantages did each online platform have? Do you have any suggestions to improve the course climate?
7. In this course, you were assessed by various tools. (GRFs, OSCE, MCQ exam, clinical reasoning test, etc.) What did you think about each one? What were the advantages and disadvantages of using each tool? What suggestions do you have for improving the evaluation of trainees?
8. How did this course prepare you to attend the in-person rotations as a medical student? During your rotations in the hospitals, what experiences did you have that the learnings from this course helped you? What experiences did you have that made you feel the need for more training? (in the form of e-learning)
9. What is your perception on skill training sessions (communication skills, reflection, clinical reasoning, medical documenting)? Were you able to apply these teachings in practice? What challenges did you have? What suggestions do you have to improve their online training?
10. In general, what is your attitude towards online education? Please explain.

- Is there anything you would like to add? What suggestions do you have to improve this course in the future?

Thank them again for their cooperation, and provide them with your contact info, in case of further concerns or questions.

**Interview questions**

1. How many sessions did you teach in this course? How do you remember those sessions in general?
2. What do you think are the most important strengths and weaknesses of online teaching? What was the best/worst thing about your class(es)? Please explain.
3. In general, what is your attitude towards online education? Has teaching in this course had an impact on your view of e-learning? Please explain.
4. In your opinion, how is the attitude/performance of these students? Have you felt a difference compared to the previous groups (who took the classes in person)? Please explain.
5. Do you prefer to use synchronous or non-synchronous online teaching, or a combination of both? Why?
6. There are various methods and tools for virtual teaching. (Lecture, case discussion, etc. / PowerPoint, the movie, Prezi, etc.) How do you feel about each one and which one do you prefer? Why? Please explain.
7. In case of technical problems, did you receive the necessary support from the organizers? Please explain.
8. What unexpected, negative or positive consequences and results has the virtualization of the classes had for you?
9. Is there anything else you would like to add?
10. (After summarizing the interview’s main points) How can we improve the quality of these classes? What suggestions do you have?

Thank them again for their cooperation, and provide them with your contact info, in case of further concerns or questions.
